# In Vitro Chondrogenic Differentiation of Human Adipose-Derived Stem Cells by Diacerein

**DOI:** 10.5812/ijpr-137803

**Published:** 2023-10-28

**Authors:** Ali Honarpardaz, Morteza Daliri Joupari, Sajjad Tavakkoli

**Affiliations:** 1 Department of Animal Biotechnology, National Institute of Genetic Engineering and Biotechnology, Tehran, Iran; 2 Gastroenterology and Liver Diseases Research Center, Research Institute for Gastroenterology and Liver Diseases, Shahid Beheshti University of Medical Sciences, Tehran, Iran

**Keywords:** Adipose Tissue, Mesenchymal Stem Cells, Cartilage Disease, Chondrogenesis, Diacerein

## Abstract

**Background:**

Tissue engineering is the application system that tries to restore damaged tissues by different approaches, such as cellular therapy, application of cell differential factors, and various materials. One of the important goals in tissue engineering is to guide stem cells directly to the desired tissue, and researchers tried to utilize different molecules as effective factors to improve this technique.

**Objectives:**

This study aims to demonstrate the effects of diacerein, a slow-acting drug for the treatment of osteoarthritis, on mesenchymal stem cell proliferation and evaluate its potential in the chondrogenesis process.

**Methods:**

Stem cells were isolated from adipose tissue, characterized by flow cytometry, and cells were treated with 10-5M diacerein for three weeks. Chondrogenic gene expression of *SOX9, COL2A1, ACAN,* and *TGFB1* were analyzed by qRT-PCR and immunocytochemistry techniques.

**Results:**

Our results showed that diacerein increased the expression of the following genes involved in chondrogenesis: *SOX9* (2.9-fold, P < 0.00), *COL2A1* (2.2-fold, P < 0.00), *ACAN* (2.7-fold, P < 0.00), and *TGFB1* (2.6-fold, P < 0.00). Immunocytochemistry results also showed increased production of collagen type II as the main protein marker for chondrocytes.

**Conclusions:**

We observed that diacerein alone could initiate and enhance chondrogenesis, and it can be used as a differentiation factor for stem cells to chondrocyte besides its ability to inhibit IL-1β. Knowing the actual function of diacerein, it could be a good candidate for the treatment of osteoarthritis.

## 1. Background

Cartilage is an important tissue in the human body due to its actions and duties during handling attritions and pressures on joints, specifically in the knee. Cartilage is an avascular tissue and lacks self-healing and repair potential. Therefore, when cartilage is damaged for any reason, such as injury or aging, it must be repaired by medicines such as non-steroidal anti-inflammatory drugs (NSAIDs) or by replacing damaged cartilage with artificial joints (1).

Tissue engineering is a practical technique utilized as an effective tool by researchers and physicians to treat and replace damaged tissues. Researchers are trying to use a different combination of materials, cells, and factors as requirements for tissue engineering to fabricate novel treatments for different tissues needing replacement or treatment (2). Cartilage is the tissue that aging or physical injuries can damage and, because of these reasons, needs to be replaced or repaired. In the meantime, many scientists and researchers are attempting to find and use different molecules as growth or differentiation factors for specific purposes, such as differentiating mesenchymal stem cells (MSCs) from specifically targeted cells such as chondrocytes (3, 4). Although many molecules have already been discovered and used as drugs, the pathways still have not been checked as initiators for differentiation.

The most commonly identified sources for MSC isolation consist of adipose tissue, bone marrow, peripheral blood, umbilical cord blood, Wharton’s jelly, and fetal tissues. The remarkable expansion capacity of these cells in vitro allows them to quickly reach the expected number for in vivo therapy (5, 6). Due to characteristics such as greater proliferative potential, ease of access and harvest, and less invasiveness, the isolation of MSCs from adipose tissue has received more attention than bone marrow and other tissues. In addition, fibrosis reduction, vessels increase, and collagen structure modification in transplanted tissues are created due to the transplantation of adipose tissue to different organs. For this reason, they are extensively studied for defective pancreatic islet cells, tendons, and damaged articular cartilages (7-9).

Many medicines have been used for years as the standard treatment for specific diseases in different countries. Based on the new vision about medicines and small molecules, there is a lack of knowledge about the full potential of these medicines in treating other diseases and efficiency on other molecular pathways (10). Due to these visions in European countries since 1990, scientists started new research on clinically approved medicines and even the small molecules used before that time as drugs (11). These events became the new research process that tries to clarify these molecules’ effects on other diseases and pathways. This research is being done to improve the knowledge about medicines and small molecules in new efficiency and treatments for treating damaged cartilage.

For many decades, the *Cassia* gender plant has been the source of diacerein extraction. The effective metabolite of diacerein is rhein, and its action is based on suppressing the interleukin 1 beta (IL-1β) pathway and downregulating IL-1β inflammatory effects (12). Diacerein is used orally, and patients suffer from side effects such as gastrointestinal symptoms, diarrhea, and soft stools. Due to these side effects, some countries organizations restricted this drug, such as the US Food and Drug Administration (FDA) (13). Lately, scientists and researchers have been trying to find new ways to reduce the side effects of diacerein, such as changing the oral to local usage. On the other hand, some of these researchers tested the effect and function of this drug for other pathways and treatments. For example, researchers tried to demonstrate that diacerein could be used as an anti-cancer drug by its inhibitory effect on IL-1β (14). Many other roles for diacerein are still unrevealed.

Many studies have been carried out to find chemical pathways for chondrogenesis, and it has been found that growth factors have critical roles during this process. Transforming growth factor beta (TGF-β) is the main factor that induces chondrogenesis in MSCs (15). During chondrogenesis, TGF-β interceded by Smad transcription factors that bind to TGF-β receptors and regulate the expression of *SOX9* transcription factor. *SOX9* is an essential factor for chondrogenesis. Research has demonstrated the autocrine/paracrine effects of the TGF-β signaling pathway on cells during chondrogenesis and even after differentiation (16). Therefore, activating the TGF-β pathway can initiate cells to start the chondrogenesis process (17). This process is associated with the expression of specific cartilage genes, which consist of genes for cartilage extracellular matrix ingredients, particularly collagen type II 1 (*COL2A1*), aggrecan (*ACAN*), and cartilage matrix protein, in different induction kinetics. Mediators of chondrogenic differentiation and cartilage development include transforming growth factor beta (TGFβ), sex-determining region Y-box 9 (*SOX9*), and runt-related transcription factor 2 (*RUNX2*). The regulation of the expression of these factors and genes is done at the transcription level, spatially and temporally, so that during chondrogenesis, they have distinct and dynamic expression patterns (18, 19).

Previous studies have demonstrated that the expression of TGF-β was increased by diacerein when added to the chondrocyte culture, but this was never examined on MSCs for chondrogenesis (20). This study aimed to show the effects of different concentrations of diacerein on MSC proliferation during cell culture and scrutinize diacerein’s potentiality on the chondrogenesis process.

## 2. Objectives

This study aimed to show the effects of different concentrations of diacerein on MSC proliferation during cell culture and scrutinize diacerein’s potentiality on the chondrogenesis process.

## 3. Methods

### 3.1. Materials

Diacerein (≥ 95% HPLC), Alcian blue, trypsin, Ascorbic acid, dexamethasone, 3-(4,5-dimethylthiazol2-yl)-2,5-diphenyltetrazolium bromide (MTT) powder, Oil Red-o, Alizarin Red-s, glutaraldehyde, and dimethyl sulfoxide (DMSO) were purchased from Sigma-Aldrich (Germany). Dulbecco’s Modified Eagle’s medium (DMEM), phosphate buffered saline (PBS), and fetal bovine serum (FBS) were purchased from Gibco (USA). The real-time SYBER Green RT-PCR was purchased from Yektatajhiz, Iran.

### 3.2. Mesenchymal Stem Cells Isolation from Adipose Tissue

Human fat was harvested from donor abdomens in six distinct areas, typically through a 2 mm-diameter cannula, into a 10-cc syringe. The samples were first washed in normal saline and then collected in (1X) PBS containing antibiotic (Penicillin and Streptomycin) solution (1 mL/100 mL). Samples were dispensed into sterile culture plates and minced in (1X) PBS using sterile surgical blades and forceps. They were then transferred into 15 mL corning centrifuge tubes containing 0.2% collagenase type I enzyme (Sigma-Aldrich) and kept for digestion in a water bath at 37°C for 20 minutes. The solution was vortexed every 2 minutes till the solution appeared clear. DMEM with 10% FBS was added to neutralize the collagenase enzyme. The sample was then centrifuged at 520 g for 10 minutes. The upper layer of the adipose tissue was discarded. The lower liquid was suspended in an equal volume of 0.2% Collagenase type I and incubated for 20 minutes in a water bath for 10 minutes. The solution was again centrifuged at 520 g for 5 minutes, and the adipose-derived stem cells (ADSCs) pellet was resuspended in complete media containing 10% FBS and 1% antibiotic solution. Seeded culture flasks were incubated at 37°C in a CO_2_ incubator with 5% CO_2_ (21).

### 3.3. Characterization of Stem Cells

#### 3.3.1. Flow Cytometry

Flow cytometry was done to characterize isolated cells. Briefly, the cultured cells were trypsinized with 0.05% Trypsin/EDTA and washed with PBS containing 2% FBS. To detect mesenchymal markers, cells were incubated with monoclonal antibodies (CD73, CD90, CD44, CD105, and hematopoietic markers CD45, CD34) (BD Biosciences Cat# 562245) for 30 minutes at 4°C, whereas conjugated isotype controls were used as negative controls. After incubation, cells were washed three times for 5 minutes each with PBS containing 2% FBS, then analyzed with the FloMax software (FloMax, version. 2.8) (22).

#### 3.3.2. Adipogenic and Osteogenic Differentiation

Differentiation of isolated ADSCs to adipocytes and osteocytes was done to show the potential potentiality of the cells. Cells were seeded in 24-well plates with a density of 3 x 10^3^ cells in 1.5 mL culture media. Plates were incubated at 37°C with 90% humidity and 5% CO_2_ for 24 hours. After 24 hours, cells were washed with PBS and replaced with adipogenic and osteogenic differentiation media (Gibco). Differentiation media was changed every week for 28 days. While monitoring cell growth, Oil Red-o Staining for adipose cells was performed at 21 days, and Alizarin Red-s staining was done for osteocytes at 28 days. Cells were fixed with 2.5% glutaraldehyde and incubated for 30 minutes at room temperature in a dark place. Then glutaraldehyde was discarded, and cells were washed with PBS. A saturated stock of Oil Red O (0.25 - 0.5%) was added to fixed cells for 10 minutes, removed, and washed with ddH_2_O three times. Alizarin Red S (2%) was added to the fixed cells and incubated at room temperature in the dark for 45 minutes. Then, Alizarin Red S solution was removed and washed three times with 1.5 mL ddH_2_O.

### 3.4. Cytotoxicity and Viability

3-(4,5-dimethylthiazol2-yl)-2,5-diphenyltetrazolium bromide assay was used to measure cell toxicity and viability of diacerein. Characterized MSCs were seeded in a 96-well plate with 10^4^ cell/well density. Plates were incubated at 37°C with 95% humidity and 5% CO_2_ for 24 hours. 3.68 mg of diacerein was weighed and dissolved in 1 mL of DMSO as a solvent to prepare the 10 mM stock solution. Then, to reach 10 - 4 M, 100 λ from stock was diluted in 10 mL of DMEM and utilized for tests. For 10^-5^ M preparation, 1 mL of 10^-4^ M prepared medium was diluted in 9 mL of fresh DMEM, and for 10^-6^ M, the process was repeated, and 1 mL media from 10^-5^ M solution was added to 9 mL of DMEM. After 24 hours of incubation, the media was refreshed by new media (DMEM medium with 10% FBS) containing different concentrations of diacerein (10^-4^ M, 10^-5^ M, and 10^-6^ M). Negative controls only received media without diacerein. After 72 hours of treatment, the media was replaced with DMEM containing 10% FBS and 10% MTT (5 mg/mL) in all the wells. After four hours of incubation, the media was removed from the wells, and 50 μL of DMSO was added to each well. After 30 minutes, the optical density of formazan was measured spectrophotometrically at 570 nm using an ELISA plate reader (BioTek EL × 800). This absorbance value is proportional to the number of viable cells. Each of the specimens was plated in triplicate (23).

### 3.5. Assessment of MSCs Chondrogenic Differentiation Potential

#### 3.5.1. Diacerein Treatment

MTT assay showed diacerein at 10^-5^ M concentration as the highest viability with the least cytotoxicity was chosen for differentiation study. Human adipose-derived stem cells (HADSCs) with a density of 3×10^4^ were seeded into the 24-well cell culture plate and incubated at 37°C with 95% humidity and 5% CO_2_ for 24 hours. After incubation, the media was changed with differentiation media (DMEM, 10% FBS, and 10^-5^ M diacerein) and incubated under the same condition for three weeks. The media was refreshed twice a week. In the control group, HADSCs were cultured in media without diacerein. All the tests were performed in triplicate.

#### 3.5.2. Alcian Blue Staining

Cells in the wells were fixed by 1% glutaraldehyde for 20 minutes, and then Alcian Blue solution was added to each well. After 30 minutes, the solution was removed, and wells were first washed with 0.1 molars of hydrochloric acid, followed by three times washing with DPBS. The samples were visualized by a Nikon 100 inverted microscope (23).

#### 3.5.3. Quantitative Real-time Reverse Transcription-Polymerase Chain Reaction

Differentiated chondrocyte cells by diacerein were validated by quantitative real-time reverse transcription-polymerase chain reaction (qRT-PCR). RNAs from HADSCs were extracted using an RNA extraction kit (Ribo Ex LS, GeneAll, Korea) according to the manufacturer’s protocol. The RNA samples were treated with DNase to eliminate possible residues of genomic DNA and reversely transcribed to cDNA by cDNA synthesis kit (Arya Tous, Iran). The qRT-PCR was performed using cDNA as the template in a 20 μL reaction mixture containing RT-PCR YTA SYBR Green. *SOX9*, *COL2A1*, *ACAN*, *RUNX2*, and *TGFB1* are specific chondrogenic genes, and GAPDH is the reference gene. The primers of these genes were designed by Oligo 7 software and verified by NCBI Primer Blast. Table 1 shows the designed primers with specifications. Results were analyzed by the software supplied with the ABI Step One real-time PCR system machine (Thermo Fisher Scientific). Gene expressions were calculated relative to GAPDH and controls by the fluorescence threshold of the amplification reaction and the comparative CT method (24).

**Table 1.** Primer Sets for Quantitative RT-PCR Related to Gene Expression Analysis

Genes and Primers	Annealing Tm°C	NCBI Accession Number	Amplicon Size
***SOX9***	59	NM_000346.4	176
F: 5’-CTAAAGGCAACTCGTACCCAA-3’			
R: 5’-GATTCTCCATCATCCTCCACG-3’			
***COL2A1***	60	NM_001844.5	138
F: 5’-CCTCTGCGACGACATAATCTG-3’			
R: 5’-TCTCCTTTCTGTCCCTTTGGT-3’			
***ACAN***	62	NM_001369268.1	182
F: 5’-TGCGGGTCAACAGTGCCTATC-3’			
R: 5’-CACGATGCCTTTCACCACGAC-3’			
***RUNX2***	59	NM_001015051.4	105
F: 5’-TAGGCGCATTTCAGGTGCTT-3’			
R: 5’-TGCATTCGTGGGTTGGAGAA-3’			
***TGFB1***	60	NM_000660.7	121
F: 5’-ATTTGGAGCCTGGACACGCA-3’			
R: 5’-AGTACACGATGGGCAGCGG-3’			
***GAPDH ***	64	-	-
F: 5’-CCATCACTGCCACCCAGAAGAC-3’			
R: 5’-GATGACCTTGCCCACAGCCTTG-3’			

#### 3.5.4. Immunocytochemistry

Immunocytochemistry (ICC) was performed to detect the production of specific chondrocyte proteins by differentiated HADSCs. Collagen type II was selected as a chondrogenesis marker. Culture HADMCs with 10^-5^ M diacerein treatment were at 37°C with 95% humidity and 5% CO_2_ for 3 weeks. Cells were fixed by 4% paraformaldehyde (Sigma-Aldrich) at 4°C for 30 minutes. Paraformaldehyde was discarded, and cells were washed with PBS twice. A blocking agent (5% goat serum) was added to each well for 45 minutes. After blocking, Collagen type II primary antibody (Abcam, Cat# ab34712) was added to each well and restored at 4°C overnight. Cells were washed with PBS, and the secondary antibody (Proteintech, Cat# SA00001-1) was added and incubated for 1 hour at 37°C condition. In the end, the wells were washed with PBS-TWEEN solution. DAPI (Sigma-Aldrich) staining was also done simultaneously parallel to the ICC assay to show the morphological characteristics of the differentiated cells (24).

### 3.6. Statistical Analysis

The results were performed from triplicate studies. SPSS software (IBM SPSS Statistics) version 23 was used as the analysis system and evaluated by one-way ANOVA with a Tukey alpha (P < 0.05). qRT-PCR results were evaluated by one-way ANOVA with a Tukey's post hoc test using 2^-ΔΔ^ Ct value for each gene. Statistical significance was considered when the P-value < 0.05.

## 4. Results

### 4.1. Isolated MSCs Characterization

**Figure 1.**
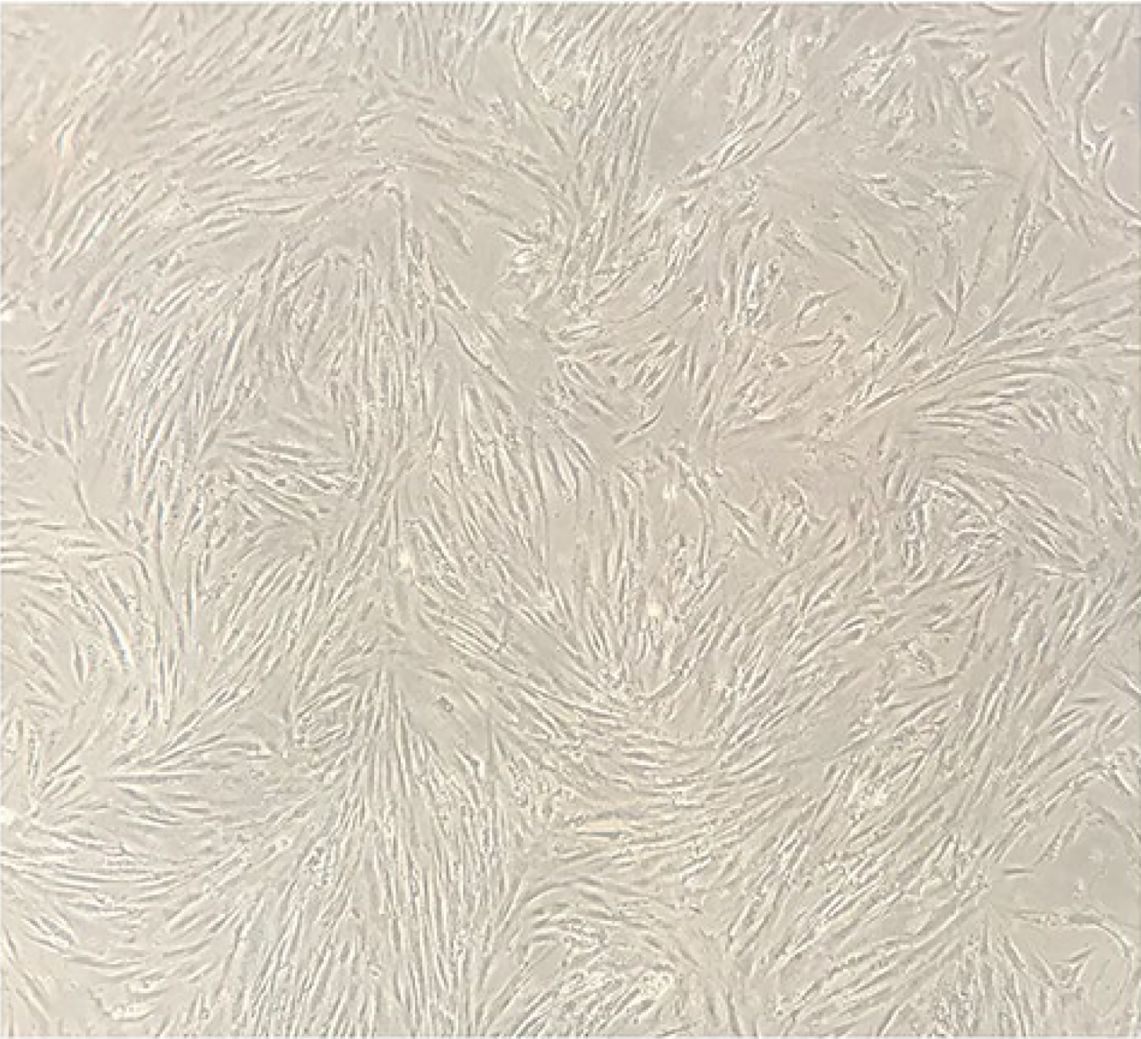
Microscopic image of isolated HADSCs after three passages (20X)

**Figure 2.**
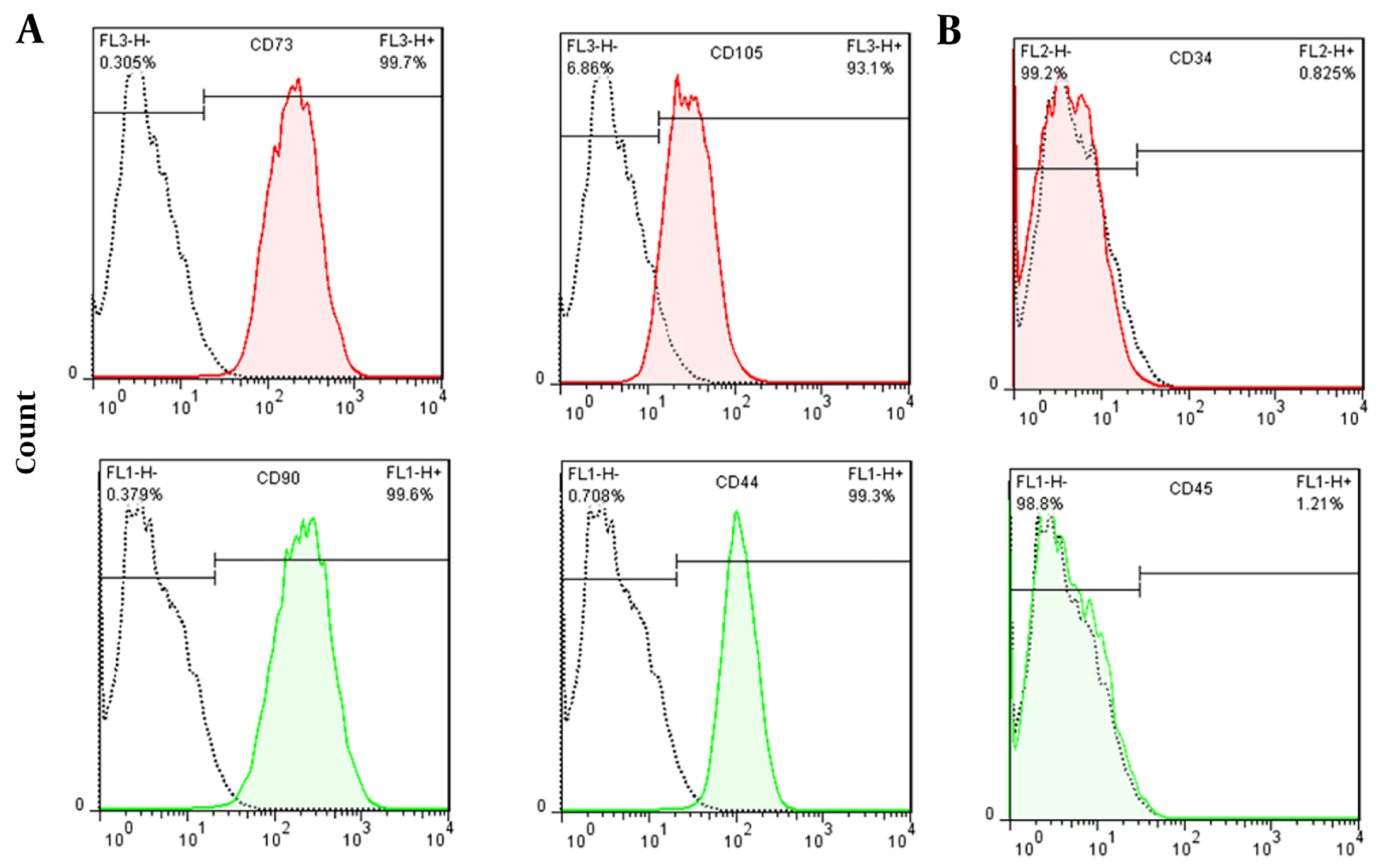
Flow cytometry results of extracted HADSCs. (A) Positive markers CD73 (99.7%), CD90 (99.6%), CD44 (99.3%) and CD105 (93.1%); (B) Negative markers CD34 (0.8%) and CD45 (1.2%)

**Figure 3.**
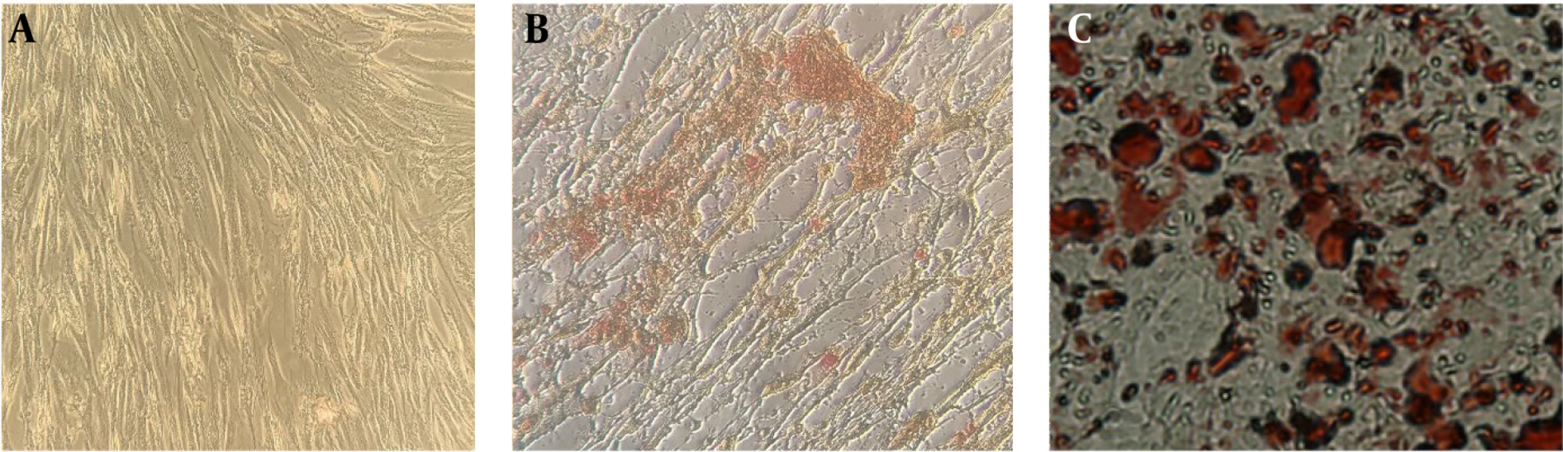
Results of osteogenic and adipogenic process. (A) Control, (B) Alizarin Red S staining for osteogenic process, (C) Oil Red O staining for adipogenic process (20X)

Figure 1 shows isolated primary mesenchymal cells, and Figure 2 shows the result for flow cytometry of isolated cells. Stem cells obtained in the third passage of cell culture were examined to confirm the expression of surface markers. Flow cytometry analysis displayed that these cells positively expressed MSC markers CD73 (99.7%), CD90 (99.6%), CD44 (99.3%), and CD105 (93.1%), whereas their expression was negative for hematopoietic markers CD34 (0.8%) and CD45 (1.2%). Adipogenic and osteogenic processes were visualized through Oil Red O and Alizarin Red staining. Figure 3 shows differentiated isolated stem cells to adipose and osteocyte cells. Differentiation into osteocytes was confirmed by extracellular matrix mineralization of differentiated cells in the presence of Alizarin Red (Figure 3B). In addition, the accumulation of lipid droplets in cells indicated the adipogenic differentiation of stem cells by Oil Red O staining (Figure 3C). There was no difference in the staining of control cultures in normal growth media (Figure 3A). 

### 4.2. Cytotoxicity and Viability

**Figure 4.**
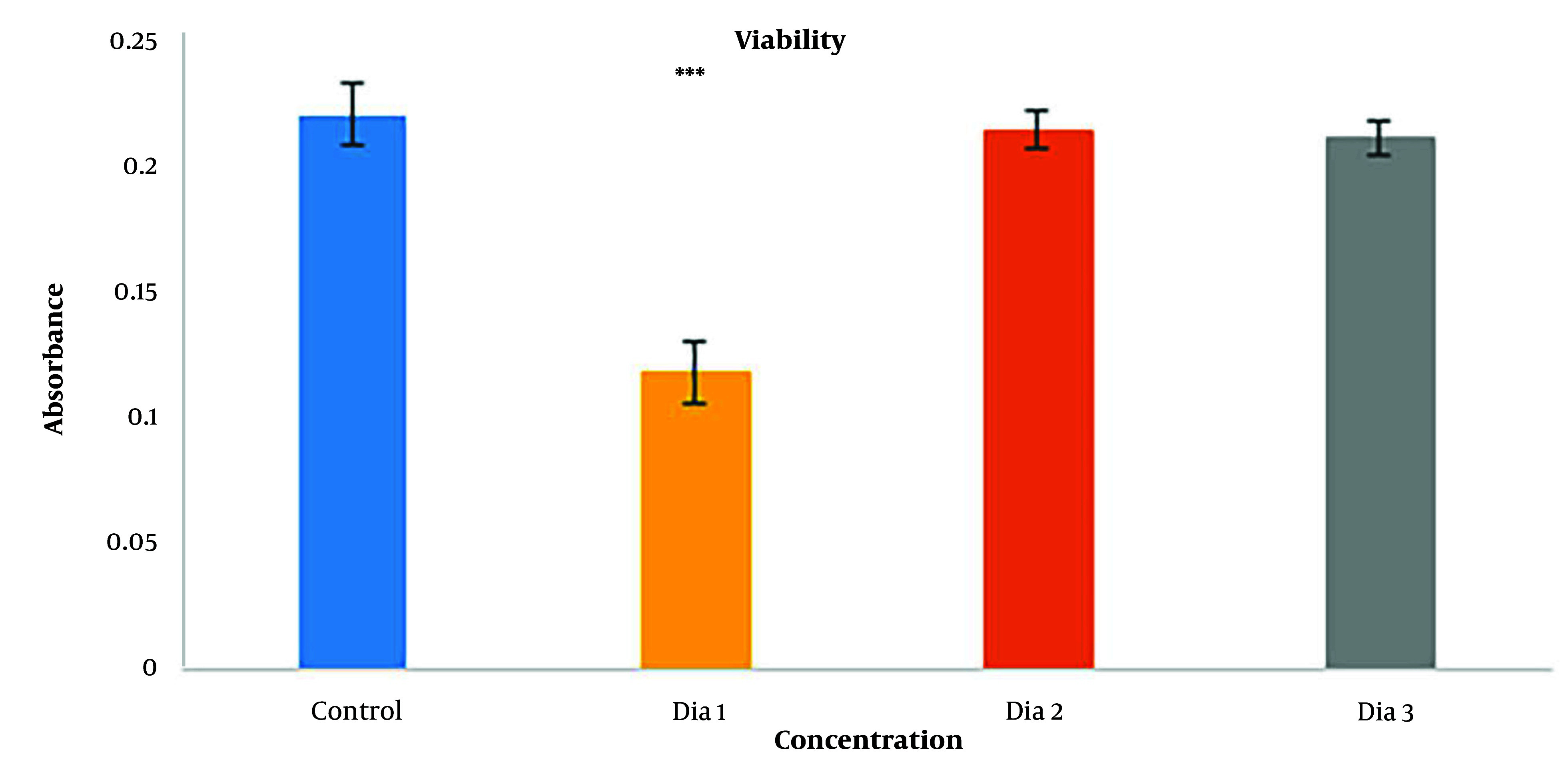
Viability results of HADSCs cells after 72 h by MTT assay (*** = P < 0.001). (dia 1) Diacerein with 10^-4^ M concentration compare to control (P < 0.001), (dia 2) diacerein with 10^-5^ M concentration compare to control (P = 0.865), (dia 3) diacerein with 10^-6^ M of diacerein compare to control (P = 0.679)

MTT assay was done to check the cytotoxicity of diacerein. Three concentrations were chosen as the trial test: 10^-4^ M, 10^-5^ M, and 10^-6^ M—the Highest nontoxic concentration of DMSO as the solver was 1% (25). Figure 4 shows the results for the MTT assay, and as demonstrated, the 10^-4^ M concentration (dia 1) was toxic for cells compared to the controls, with a highly significant difference (P < 0.001). Diacerein with 10^-5^ M concentration (dia 2) was the highest concentration that was not toxic for cells with no significant difference to control (P = 0.865), and also diacerein with 10^-6^ M (dia 3) (P = 0.679) had no cytotoxicity effect on cells. 10^-5^ M concentration was chosen as the highest nontoxic concentration for the chondrogenesis study.

### 4.3. Chondrogenesis Study

#### 4.3.1. Alcian Blue Staining

**Figure 5.**
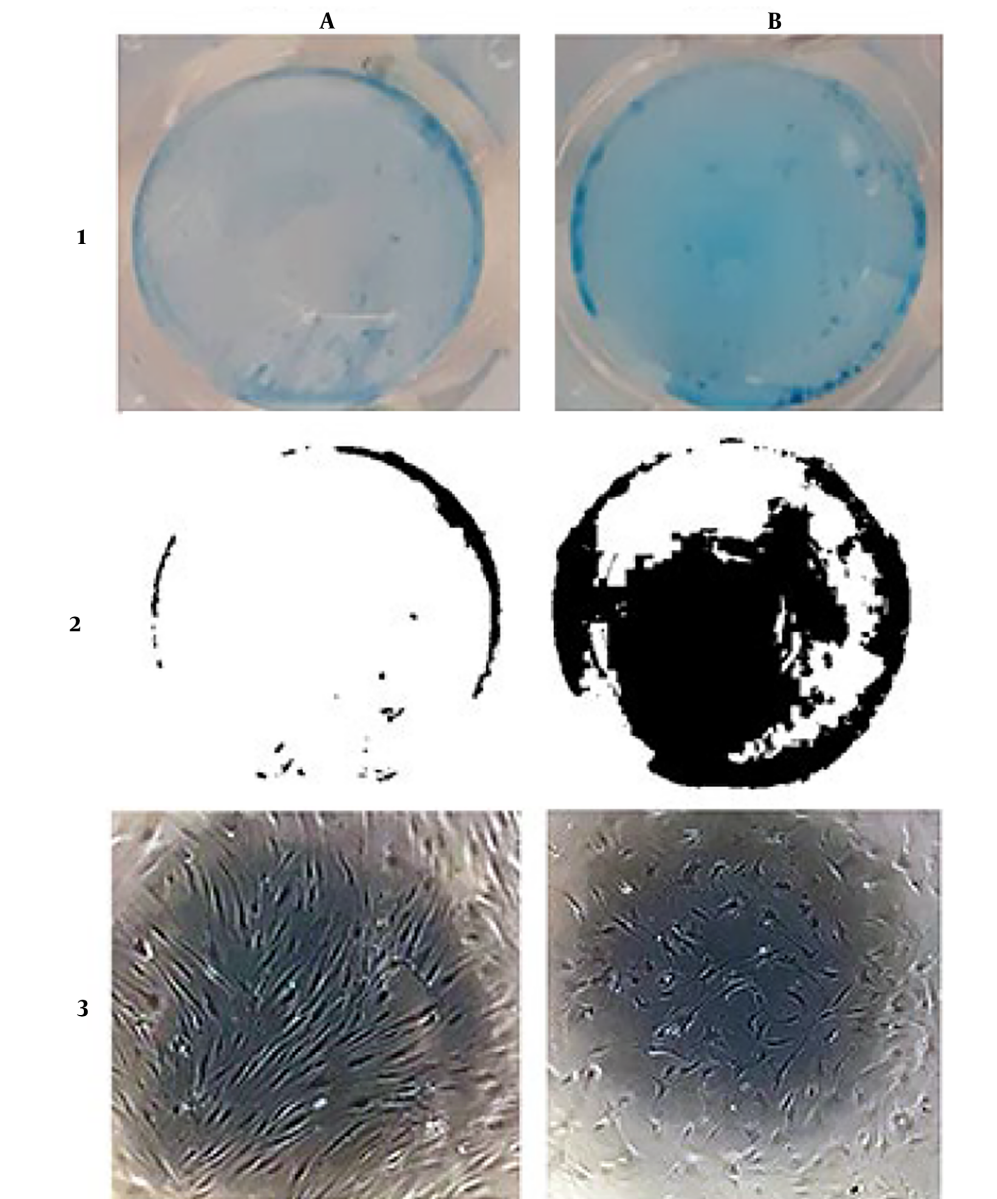
Alcian blue staining of cells after 3 weeks. (A) Control cell, (B) treated cells with Diacerein 10-5 M. (1) Alcian blue stain on cells, (2) threshold of stained cells by Image J software, (3) morphology of cells after 3 weeks

Alcian Blue showed the amount of GAGs produced by cells. Figure 5 shows Alcian Blue staining of the cells after 3 weeks of incubation with diacerein compared to the control group. Blue stain represents the GAGs that are produced and released by cells. Treated cells with diacerein showed increased severity of blue color compared to control cells.

#### 4.3.2. RT-PCR

**Figure 6.**
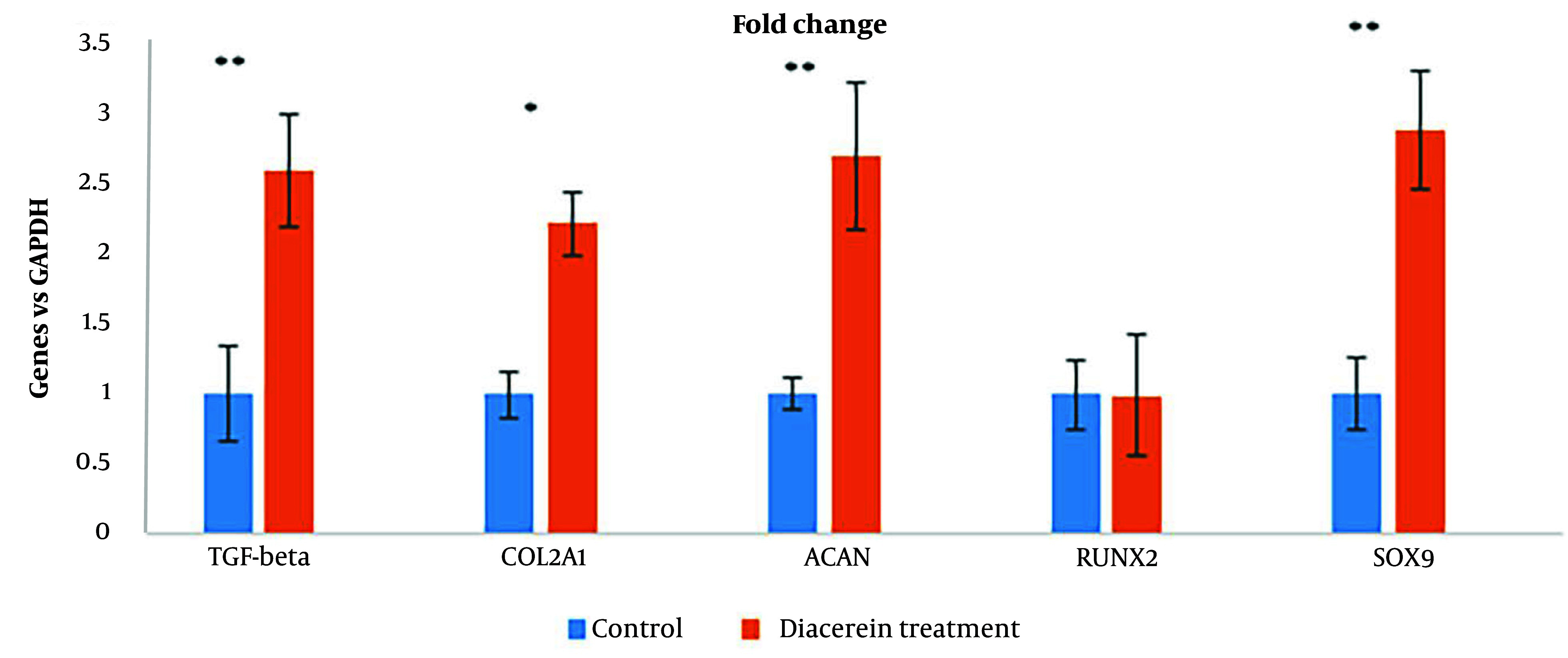
qRT-PCR analysis of genes by one-way ANOVA (*P < 0.05) (**P < 0.00). *SOX9* (2.9 fold, P < 0.001), *COL2A1* (2.2 fold, P < 0.001), *ACAN* (2.7 fold, P < 0.001), *TGFB1* (2.6 fold, P < 0.001) and *RUNX2* gene expression (P < 0.1)

Gene expression during chondrogenesis demonstrated the potentiality of diacerein. Figure 6 shows the effect of diacerein with 10^-5^ M concentration on chondrogenesis gene expression: *SOX9* (2.9 fold, P < 0.001), *COL2A1* (2.2 fold, P < 0.001), *ACAN* (2.7 fold, P < 0.001), *TGFB1* (2.6 fold, P < 0.001) and *RUNX2* gene expression (P < 0.1) did not show significant difference.

#### 4.3.3. Immunocytochemistry

**Figure 7.**
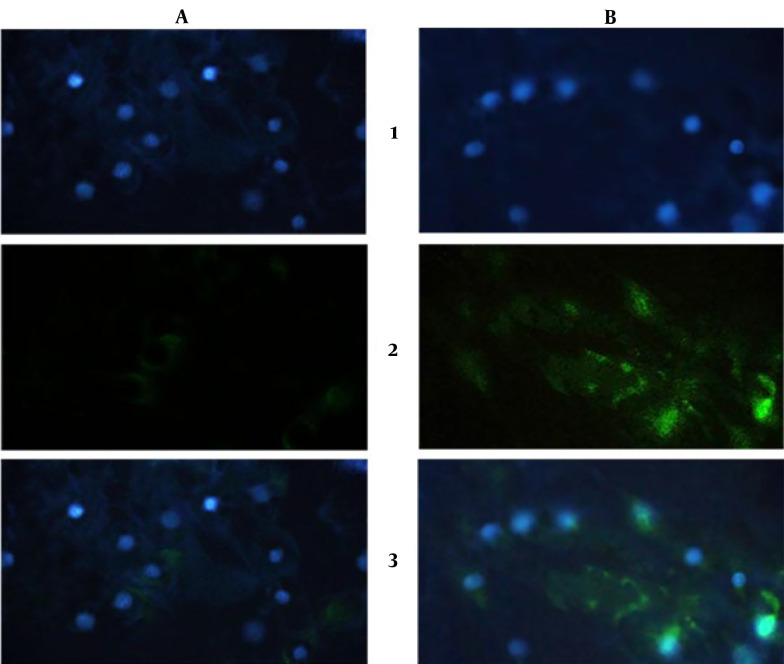
ICC results after a three-week cell differentiation test. 1- DAPI staining: (A) control, (B) diacerein treatment; 2- Collagen type II expression: (A) control, (B) diacerein treatment; 3- Merged picture of DAPI and collagen type II expression. (The green color shows the collagen type II, and the blue color shows the cells nuclei)

Visualization of expressed collagen type II protein in ICC test and DAPI staining (Figure 7) showed expression of collagen type II was increased (B-2) compared to control (A-2), and cell nuclei were healthy during the test (A-1 and B-1) after 3 weeks.

## 5. Discussion

Diacerein is a drug prescribed for osteoarthritis in many countries due to its therapeutic properties, such as anti-inflammatory, anti-catabolic, and pro-anabolic actions during cartilage healing, by inhibiting the IL-1β signaling pathway. However, when administered orally, diacerein was restricted because of its side effects, mainly diarrhea (26).

Defects in cartilage tissue cause many health problems, and tissue engineering is a practical technique for its repair by utilizing chondrocytes that differentiate from stem cells. This study aimed to present diacerein as a new differentiation factor for chondrogenesis of human mesenchymal stem cells during the application of tissue engineering (27).

Researchers tried to enhance the effects and reduce the side effects of diacerein. This study is the first step to show that the side effects of diacerein can be minimized by changing its application methods to local usage as a differentiation factor for the chondrogenesis process in tissue engineering technique while retaining its inhibitory beneficial properties (28, 29).

Cells are an inseparable part of tissue engineering; stem cells play a key role during differentiation studies. HADMCs were chosen as the primary cell type for this study due to easy isolation and multipotency to differentiate from other types of cells (30). During this study, HADMCs were differentiated into chondrocytes, indicating that this type of cell has a high potential to be utilized for chondrogenesis study.

Diacerein was optimized by MTT assay. Diacerein at a maximum concentration of 10^-4^ M was dissolved in 1% v/v DMSO (31) while DMSO did not show any toxic effect on cellular proliferation, whereas diacerein at 10-4 M showed high cellular mortality; therefore, 10^-5^ M diacerein with low toxicity and high proliferation was the chosen concentration (32).

Alcian Blue stains GAGs during chondrogenesis. HADMCs treated cells with diacerein gained more stain by Alcian blue compared to control cells. Diacerein could increase cell GAG production, a sign of chondrogenesis (33).

TGF- β is one of the main factors during chondrogenesis, by initiating and inducing expression of its targeted genes such as *SOX9* and *COL2A1*. Based on previous studies, diacerein could increase the gene expression that interferes with collagen production by activating the TGF-β pathway in chondrocytes (16, 34). By increasing the expression of TGF- β, diacerein makes cells express the chondrogenesis genes and differentiate into chondrocytes. Differentiated cells produce specific proteins released into the environment around cells. In this study, mesenchymal cells were differentiated into chondrocytes using diacerein and blue stain, indicating that GAGs were released by differentiated cells. Diacerein could enhance the expression of TGF-β in chondrocytes (20), and it is demonstrated that TGF-β can initiate chondrogenesis in mesenchymal stem cells by activating other signaling pathways (35). Specific genes such as *SOX9* are activated by TGF-β to control chondrogenesis (36). One of the important regulator proteins in chondrogenesis is *SOX9*, encoded by the *SOX9* gene. *SOX9* protein increases the expression of other genes like *COL2A1* and *ACAN*. In this study, we showed that the expression of *SOX9* was increased by 2.9-fold when 10^-5^ M diacerein was added to the media as a differentiation factor, and it also increased *COL2A1* and *ACAN*. *COL2A1* and *ACAN* are the specific genes during chondrogenesis since they make proteins essential for cartilage production by chondrocytes (37). It also has been suggested that an increase in the tissue inhibitor of metalloproteinase-1 (TIMP-1) and a decrease in the generation of pro-matrix metalloproteinases (MMPs), which play a role in cartilage destruction, are caused by diacerein at a gene expression level (38, 39).

During the differentiation of stem cells to chondrocytes, there is a great concern that the cells may not differentiate into osteoclasts. A previous study showed that diacerein can block osteogenesis by inhibiting IL-1β (40). Several studies have shown that diacerein's efficacy reduces IL-1β-induced inflammatory pathways, mainly in cartilage degradation (16, 41). The mechanism of chondrogenesis and osteogenesis is controlled by different expressions of *SOX9* and *RUNX2* genes (33). *SOX9* is a key transcriptional activator of chondrogenesis, and its increased expression in cells induces differentiation into chondrocytes. On the other hand, by elevating the expression of *RUNX2*, a main transcription factor for osteogenesis, the cells will be guided to the osteogenesis path (42). Our findings showed that the expression of *SOX9* was gained 2.9-fold, while there was no significant difference in *RUNX2* expression. Collagen type II is the base product of the chondrocytes and can be considered an important marker protein for cartilage (43). The *COL2A1* gene is translated to one part of the collagen type II protein, and by an increase in the expression of *COL2A1*, the production of collagen type II also increases (44). As we demonstrated, expression of *COL2A1* was increased around 2.2-fold, and in ICC assay, production of collagen type II was also increased after three weeks. Altogether, our findings were consistent with previous reports demonstrating diacerein's potential in promoting chondrogenic differentiation by up-regulating the expression regulator proteins during chondrogenesis (*COL2*, *SOX9*, and *ACAN*) (34, 45).

### 5.1. Conclusions

This study showed restricted drugs may be used as a molecular signaling pathway enhancer or initiator. Evidence in this paper proved that a specific concentration of diacerein can initiate chondrogenesis without using any other factors or special media and without any toxic effects on cells in the specific range. With this differentiation potentiality and capability to inhibit the IL-1β pathway as the main factor for cartilage defections, diacerein can be a suitable candidate for treating damaged cartilage by tissue engineering techniques.

## Data Availability

Data supporting this article are available from the corresponding author upon reasonable request.
